# Birth preparedness and complication readiness among pregnant women in Tehulederie district, Northeast Ethiopia: a community-based cross-sectional study

**DOI:** 10.1186/s12912-018-0278-y

**Published:** 2018-03-15

**Authors:** Demlie Belete Endeshaw, Lema Derseh Gezie, Hedija Yenus Yeshita

**Affiliations:** 1 Maternal and Child Survival Program (MCSP), Community Based Newborn Care (CBNC) Coordinator, Save the children, Woldia, Ethiopia; 2 Department of Epidemiology and Biostatistics, Institute of Public health, University of Gondar, Gondar, Ethiopia; 3 Department of Reproductive Health, Institute of Public health, University of Gondar, Gondar, Ethiopia

**Keywords:** Birth preparedness, Complication readiness, Knowledge, Danger signs, Northeast Ethiopia

## Abstract

**Background:**

Motherhood is a time of anticipation of joy for a woman, her family, and her community. In spite of this fact, it is not as enjoyable as it should be because of numerous reasons. Insufficiency or lack of birth preparedness and complication readiness is the most common reason. The aim of this study was to assess the practice of birth preparedness and complication readiness and associated factors among pregnant women in Tehuledere district, northeast Ethiopia.

**Methods:**

A community-based cross-sectional study was conducted in Tehuledere district, northeast Ethiopia. Participants were selected using the multistage sampling technique, and data were analyzed both descriptively and analytically using the binary logistic regression.

**Result:**

Out of the total 507 samples, 500 (response rate 98.6%) pregnant women participated in the study. Less than half (44.6%) and (43.4%) of the respondents had knowledge and practice on birth preparedness and complication readiness, respectively. In the multivariate analysis, knowledge of birth preparedness and complication readiness (AOR = 1.648, 95%CI: 1.073, 2.531), knowledge of danger signs during pregnancy (AOR = 2.802, 95% CI: 1.637, 4.793), gestational age (AOR = 3.379, 95% CI: 2.114, 5.401), and antenatal care follow up starting time (AOR = 2.841, 95% CI: 1.330, 6.068) were significantly associated with the practice of birth preparedness and complication readiness, but pregnant women in rural areas (AOR = 0.442, 95% CI:0.244, 0.803) were less associated with birth preparedness and complication readiness compared to women in urban settlements.

**Conclusion:**

This study identified that poor knowledge, inadequate birth preparedness, and complication readiness were prevalent among mothers in the study area. Government officials, partners, and health care providers working in the areas of maternal and child health should operate together to maximize birth preparedness and complication readiness practices.

**Electronic supplementary material:**

The online version of this article (10.1186/s12912-018-0278-y) contains supplementary material, which is available to authorized users.

## Background

Motherhood is a time of expectation and joy for a woman, her family, and her community. However, for thousands of women, the experience is not as pleasurable an event as it should be [1].

Each year in Africa 30 million women become pregnant, and about 250,000 of them die from pregnancy-related causes [2]. It is not possible to predict which women will experience life-threatening obstetric complications that lead to maternal mortality. On the whole, 42% of all pregnancies suffer complications – in rich and poor countries alike – and in 15% of all pregnancies, the complications are life-threatening. Scaling up birth preparedness and complication readiness (BPCR) practices and providing care by skilled providers (doctor’, nurse’ or midwives) during pregnancy, childbirth, and in the immediate postnatal period are fundamental for maternal health [2].

In many societies in the world, cultural beliefs and lack of knowledge of BPCR inhibit in advance preparations for delivery and expected babies. Since no action is taken prior to delivery, the family tries to act only when labor begins. To this end, the utilization of BPCR is the cornerstone for most developing countries, including Ethiopia. It is a strategy to promote a timely use of skilled maternal and neonatal care, especially during pregnancy, childbirth, and through the two-day post-partum period. The role of BPCR is to improve the use and effectiveness of key maternal and neonatal services through reducing the deadly delays (the first two delays) in deciding to seek care. Due to increases in the level of knowledge about complication readiness and the recognition of danger signs, the identification of problems and the delays in seeking health care services have been improved [3–8].

The Ministry of Health (MOH) has designed & initiated the application of BPCR in different maternal and neonatal health interventions to reduce maternal and newborn morbidity and mortality in Ethiopia. Currently, the Maternal Mortality Rate (MMR) of Ethiopia is 420 per 100,000 live births. Only 15% of the births in Ethiopia are at health facilities, 14% at public and 1% at private [9, 10].

Women’s BPCR practices were examined by different authors who no doubt used sound methods. For instance, a study conducted in India on BPCR among slum women recognized steps; like identifying a trained birth attendant, selecting a health facility, arranging transport for delivery and obstetric emergency, and saving money for delivery; responding positively to at least three of the four elements was considered as an appropriate preparation [11].

In community-based cross-sectional studies conducted by Mpwapwa district of Tanzania, Plateau district in Nigeria, and a rural district of Ghana on BPCR among women showed that only a few of the women knew three or more obstetric danger signs [12–14].

According to a study conducted in rural Uganda, 52% of women knew at least one key danger sign during pregnancy, 72% during delivery and postpartum periods. Overall, 35% of the respondents who made arrangements in three of the four birth preparedness practices were classified as “well prepared for birth” [15].

A similar study conducted by Ife Central Local Government, Nigeria, on BPCR among pregnant women showed that 158 (39.3%) respondents knew no danger signs of pregnancy, childbirth, and postpartum periods. Only 24 (6.0%) had adequate knowledge of obstetric danger signs without prompting. Three hundred and forty (84.8%) of women identified birthplaces and 312 (78.3%) began saving money for delivery. However, 304 (79.4%) of the women made no arrangements for blood donors [16]. Other studies conducted in Adigrat and Goba districts of Ethiopia showed that only 118 (22.1%) and 128 (29.9%) of the total respondents, respectively, were found to be well prepared ahead of childbirth [17, 18].

A study done in Aleta Wondo district using five measures of BPCR that included identifying health provider, health facility and potential blood donor, the arrangement of transportation, and saving money for costs of delivery and emergency showed that among 743 pregnant women, only 20.5% identified skilled providers. In the same study, only 8.1% identified health facilities for delivery and/or for obstetric emergencies. On the other hand, the majority (87.9%) of the respondents reported that they intended to deliver at home, whereas only 60(8%) planned to deliver at health facilities, and 17% of the pregnant women were well prepared [19].

A study on the elements of BPCR in Jimma, Ethiopia, reported that 12.5%, 65.7%, 81%, 20.2%, and 58.9% of the pregnant women made prior decisions regarding skilled birth attendance, money, place of delivery, mode of transportation, and potential blood donor, respectively. On the whole, the study identified that less than one-third, 106 (29.4%) of the study participants were prepared for BPCR, indicating the prevalence or existence of poor practices or preparedness for birth and its complications in the area [20].

With regard to risk factors, studies done in Adigrate and Goba districts showed that residence, occupation, educational level, family size, ANC follow up, knowledge of danger signs during pregnancy, labor and postnatal period, as well as gravida and parity were found to have statistically significant associations with BPCR [17, 18].

Generally, almost all of the findings in the above studies revealed that BPCR remains almost unutilized to improve maternal and neonatal health in most developing countries, especially in sub-Saharan Africa. Hence, one can clearly understand that among the population studied, BPCR is comparatively low. The findings can also tell us BPCR is very low, especially in Ethiopia, compared to findings in other developing countries. As a result, the majority of the pregnant women did not give birth at health institutions; therefore, it is necessary to further investigate knowledge and practice of BPCR and possible associated factors.

As a matter of fact, not only were studies on BPCR in this particular area scarce but also the studies available varied in terms of time and place of study, geographic location, and population. Most previous studies focused on urban communities and among non- pregnant women [17–21]. In addition, the level of BPCR practice and its associated factors among pregnant women was not clearly examined in the study areas. So, the aim of this study was to assess BPCR practices and associated factors among pregnant women in Tehulederie district, Amhara National Regional State, northeast Ethiopia. Hence, the findings of the study will be crucial for the study woreda health officials, South Wollo zone health departments, the Amhara National Regional Health Bureau, and other maternal and neonatal health program implementers.

## Methods

### Study design and setting

A community-based cross-sectional study was conducted in Tehulederie district, the Amhara National Regional State, northeast Ethiopia, 2015. The district had 134,131 inhabitants (48% female), of which 31,628 were women in the reproductive age group, and 4520 were expectant pregnant women [22]. Tehulederie has a total of 26 kebeles (the lowest administrative units usually with multiple villages). It had 21 health posts, six small private clinics, three medium private clinics, five public health centers, seven drug stores, and one diagnostic facility [23].

The study was intended to represent all pregnant women who had the chance to be sampled in the district. After orientating about the significance of the study, pregnant women able to give informed consent were included.

### Sample size determination and sampling procedure

The required sample size of the study was determined using the single population proportion formula. In determining the sample size, the proportion (p) of BPCR [18], the margin of error or precision (w), and confidence level were assumed to be 29.9%, 5%, and 95%, respectively. Moreover, a design effect of 1.5 and a non-response rate of 5% were applied to the initial sample size to get the final one (507).

In the district, about 4520 women were estimated to be eligible, that is expected to be pregnant. First, the district was stratified into urban and rural in order to deal with the effect of residence. Two of the five urban and eight of the twenty-one rural kebeles were proportionately selected by the simple random sampling technique. Then, the total sample size was allocated proportionally to each kebele according to their proportion of pregnant women. Thus, one hundred and seven participants from the two urban kebeles and four hundred from the eight rural kebeles were included in the study. To prepare the frame of pregnant mothers, Health Extension Workers (HEWs) of each selected kebele updated the identifications of pregnant women registered in the health centers/posts. Finally, every third pregnant mother was selected systematically by tracking them with the updated pregnant women registration location.

### Operational definitions

For reasons of clarity and standardization, some concepts were defined operationally. Accordingly, birth preparedness and complication readiness elements included identifying a skilled birth attendant and health facility, arranging means of transport for delivery as well as in case of obstetric emergency, saving money, arranging blood donors in case of obstetric emergency, identifying a birth companion, temporary family caregiver, medical facility in case of obstetric emergency, and decision making in case of emergency.

A woman was considered as “knowledgeable in BPCR” if she identified and mentioned at least six key components of birth preparedness and complication readiness elements [18]. Similarly, a study participant would be considered as “knowledgeable in key danger signs of pregnancy” if she could spontaneously mention at least three key danger signs of pregnancy. On the other hand, a woman would be classified as “knowledgeable in key danger signs of labor” if she could spontaneously mention at least five key danger signs of labor/childbirth. Likewise, a woman would be considered as “knowledgeable in key danger signs of postpartum” if she could spontaneously mention at least five key danger signs of a postpartum period within two days [18].

### Data collection procedure

A close-ended structured questionnaire was used to collect data from the participants. The questionnaire was prepared in English and translated to Amharic and retranslated to English by a language expert to check its consistency (Additional files 1 and 2). The questionnaire included Information on socio-demographic and obstetric characteristics, knowledge questions on key obstetric danger signs, birth preparedness, and complication readiness. After evaluation, the final version of the questionnaire was developed. For each of the questions, a participant who had the practice was given a score of “one”, and lack of the practice was given a score of “zero”. Seven diploma graduated nurses working out of the district, fluent in the local language and familiar with local norms collected the data. The data collectors interviewed participants in person. One BSc. the graduate health officer was assigned to supervise the data collection process. Training was given to both the data collectors and supervisor by the principal investigator for two days.

### Data quality control/management

In order to ensure clarity and consistency, the questionnaire was pretested on 10% of the sample in a similar population of a nearby kebele. After that, the necessary modifications were made to the items and evidence-based time was allocated to each respondent. All data collected was checked for completeness by the principal investigator and the supervisor immediately at the end of each data collection day.

### Data process and analysis

Data was entered, coded, cleaned and analyzed using SPSS version 20 software. Descriptive statistics such as frequencies and percentages were computed; then, a bivariable analysis was used to examine the crude associations of factors on BPCR. Variables with less than 0.2 *p*-values in the bivariable analysis were candidates for multivariable logistic regressions. Statistical significance was declared at a p-value of 0.05 and adjusted odds ratios were used to determine the strength of associations.

## Result

### Socio-demographic characteristics of respondents

Out of the total 507 samples, only five hundred pregnant women participated in the study, giving a response rate of 98.6%. The mean and ± Standard Deviation of respondent age was 28.8 ± 5 years; women’s age ranged from 20 to 38 years. Of the total respondents, 472 (94.4%) were married and 4.4% single. Most of the respondents (469 or 93.8%) were housewives, and 391 (78.2%) of the pregnant women were rural dwellers; 338 (67.6%) attended formal school (Table 1).

**Table 1:** Socio-demographic characteristics of pregnant women in Amhara National Regional State, northeast Ethiopia, 2015

Variables	Category	Frequency	Percentage
Age	15–24	107	21.4
	25–34	308	61.6
	35–44	85	17.0
Marital status	Single	22	4.4
	Married	472	94.4
	Divorce	4	0.8
	Widow	2	0.4
Residence	Urban	109	21.8
	Rural	391	78.2
Occupation	Housewife	469	93.8
	Government employee	9	1.8
	Merchant	15	3.0
	NGO employee	7	1.4
Religion	Orthodox	44	8.8
	Muslim	456	91.2
Family size	1–3	232	46.4
	4–6	244	48.8
	>/=7	24	4.8
Educational status	No formal education	338	67.6
	Primary education	98	19.6
	Secondary education	51	10.2
	College and above college	13	2.6
Monthly income	< 500 ETB	226	45.2
	500–1500 ETB	193	38.6
	> 1500 ETB	81	16.2
Ethnicity	Amhara	500	100.0
Partner education	No formal education	343	68.6
	Primary education	59	11.8
	Secondary education	58	11.6
	College and above College	40	8.0
Partner occupation	Farmer	354	70.8
	Employee	36	7.2
	Merchant	82	16.4
	NGO employee	28	5.6
Distance of HF from home (round trip)	</= one hour	162	33.0
	> one hour	338	67.0

### Obstetric characteristics of respondents

In the assessment of obstetric characteristics, 135 (27%) of the respondents were primigravida, and about 365 (73%) had more than two pregnancies. Regarding gestational age, 185 (37%) respondents were in the first trimester and twenty-five (5%) in the third trimester. About 358 (71.6%) respondents had two and more than two ANC visits (Table 2).

**Table 2:** Obstetric characteristics of pregnant women in Tehulederie district, Amhara National Regional State, northeast Ethiopia, 2015

Variables	Category	Frequency	Percentage
Gestational age	1–3 months	185	37.0
	4–6 months	290	58.0
	7–9 months	25	5.0
Starting time for ANC visit	No	4	.8
	Yes	496	99.2
Number of ANC visits	One time	142	28.4
	Two times	168	33.6
	Three times	135	27.0
	Four times	51	10.2
	Five times	4	.8
ANC service was given by	Midwife/clinical nurse	471	94.2
	HEWs	29	5.8
ANC starting time	at < 3 month	54	10.8
	at 3–4 month	310	62.0
	>/= 5 month	136	27.2
Plan of ANC visit	< 4 visits	14	2.8
	4 visits	471	94.2
	> 4 visits	15	3.0
Para	0	135	27.0
	1–3	227	45.4
	>/= 4	138	27.6

Regarding knowledge of key danger signs during pregnancy, childbirth, and the postpartum period within two days, 492 (98.4%) of the 500 respondents reported that they had information about danger signs during pregnancy, childbirth, and within two days of the postpartum period. About 128 (25.6%), 333 (66.6%), and 321 (64.2%) of the pregnant women were not knowledgeable about danger signs during pregnancy, labor/childbirth and within two days of the postpartum period, respectively. When vaginal bleeding, the most frequently reported obstetric danger sign was considered, 484 (96.8%) had it during pregnancy, 483 (96.6%) at labor/childbirth, and 455 (91%) in the postpartum period.

### Knowledge of respondents about preparation for birth and its complications

All of the respondents reported that they had information about BPCR. Out of the total respondents, 223 (44.6%) were considered as knowledgeable. Regarding each recommended element which had to be done as BPCR; 440 (88%) said they saved money for delivery and possible obstetric emergency, 429 (85.8%) identified place of delivery, 109 (21.8) arranged potential blood donor, and 142 (28.4) spontaneously chose a skilled birth attendant (Table 3).

**Table 3:** Knowledge of birth BPCR among pregnant women in Tehuledere district, northeast Ethiopia, 2015

Variable	Response	Frequency	Percentage
Identification of health facility	No	71	14.2
	Yes	429	85.8
Identification of skilled birth attendant	No	358	71.6
	yes	142	28.4
Saving money	No	60	12.0
	Yes	440	88.0
Preparation of transport	No	306	61.2
	Yes	194	38.8
Identification of temporary family caregiver	No	199	39.8
	Yes	301	60.2
Arranging blood donor	No	391	78.2
	Yes	109	21.8
Identification of birth companion	No	206	41.2
	Yes	294	58.8
Identification of decision-maker in case of emergency	No	207	41.4
	Yes	293	58.6
Identification of medical facility in case of emergency	No	88	17.6
	Yes	412	82.4
Knowledge about BPCR knowledgeable	No	277	55.4
	Yes	223	44.6

### Birth preparedness and complication readiness practices of respondents

The majority of the respondents reported making arrangements for some of the recommended elements of BPCR. Out of 500 participants, 391 (78.2%), 175 (35%), 336 (67.2%), 202 (40.4%), and 104 (20.8%) identified health facilities for delivery, skilled birth attendants, financial sources for delivery and possible obstetric emergencies, modes of transport, and potential blood donors, respectively. Similarly, out of the same number of participants, 352 (70.4%) identified a temporary family caregiver in case of emergency, 348 (69.6%) chose a birth companion, 380 (76%) selected medical facility in case of obstetric emergency, and 346 (69.2%) nominated decision maker in case of obstetric emergency. Overall, 43.4% of the women practiced BPCR.

### Maternal socio-demographic and obstetric characteristics associated with their practices of birth preparedness and complication readiness

In the bivariate logistic regression analysis, a statistically significant association (*p* < 0.05) was observed between maternal practices of BPCR and their educational status, gestational age, spouse occupation, knowledge of danger signs during pregnancy and childbirth, number of ANC visits, marital status, residence, spouse education, knowledge of BPCR, monthly income, and starting time of ANC service. In the multivariate logistic regression analysis, a statistically significant association was observed on knowledge of women on BPCR [AOR = 1.648, 95% CI: 1.073, 2.531]. Similarly, women knowledgeable about danger signs during pregnancy were three times more likely to practice BPCR compared to those who had no knowledge about danger signs during pregnancy [AOR = 2.802, 95% CI: 1.637, 4.793]. Pregnant women with a gestational age of 4–6 months were three times more likely to practice BPCR than women whose gestational age was up to three months [AOR = 3.379, 95% CI: 2.114, 5.401]. In addition, pregnant women who started ANC follow-up within 3–4 months of their pregnancy were three times more likely to practice BPCR compared to with those who started late [AOR = 2.841, 95% CI:1.330, 6.068] (Table 4).

**Table 4:** Maternal socio-demographic and obstetric characteristics associated with BPCR practices in Tehuledere district, northeast Ethiopia, 2015

Variables		Practices of BPCR			95% C.I. for AOR		*P*-value
		No	Yes	AOR	Lower	Upper	
Gestational age*	up to 3 month	133	52	1			
	at 4–6 month	132	158	3.379	**2.114**	**5.401**	**.000**
	at 7–9 month	18	7	1.422	.512	3.948	.500
Residence*	Urban	44	65	1			
	Rural	239	152	.442	**.244**	**.803**	**.007**
Starting time o ANC*	at < 3 month	38	16	1			
	at 3–4 month	166	144	2.841	**1.330**	**6.068**	**.007**
	>/= 5 month	79	57	1.278	.555	2.942	.564
Knowledge of BPCR*	Not knowledgeable	177	100	1			
	Knowledgeable	106	117	1.648	**1.073**	**2.531**	**.023**
Knowledge of danger signs during pregnancy*	Not knowledgeable	101	27	1			
	Knowledgeable	182	190	2.802	**1.637**	**4.793**	**.000**
Partner occupation	Farmer	229	125	1			
	Employee	12	52	2.259	.891	5.723	.086
	Merchant	32	50	2.280	1.263	4.114	.06

## Discussion

The aim of this study was to examine mainly the BPCR practice of pregnant women. Our study revealed that only less than half of the participants were practicing BPCR (217 or 43.4%) and that a nearly equal number of women were knowledgeable about the practice (223 or 44.6%).

The majority of the respondents reported that they made some arrangements on some of the recommended elements of BPCR. Out of five hundred respondents, 391 (78.2%) identified a health facility for delivery, 175 (35%) identified a skilled birth attendant, 336 (67.2%) saved money for delivery and any possible obstetric emergency, 202 (40.4%) determined mode of transport, 104 (20.8%) identified potential blood donor in case of emergency, 352 (70.4%) selected temporary family care giver, 348 (69.6%) nominated birth companion, 380 (76%) identified medical facility in case of obstetric emergency, and 346 (69.2%) selected decision maker in case of obstetric emergency. Generally, only 217 (43.4%) of the participants made BPCR arrangements.

In this study, 391 (78.2%) pregnant women identified health facility for delivery. This is almost comparable with the findings of studies done in Jimma town and Goba woreda, Oromia Region, Ethiopia, which reported 285 (81%) and 432 (76.9%), respectively [18, 20]. However, the finding of this study is lower than that of a study done in Mpwapwa district Tanzania which detected 583 (97.3%) [12]. The likely explanations for this dissimilarity might be differences in study subjects and settings. On the other hand, this finding is higher than those of studies done in Adigrat town, northern Ethiopia, and Aleta wondo, southern Ethiopia, which reported 209 (39.1%) and 59 (8.1%), respectively [17, 19]. This difference might be due to variations in the times of investigations in that currently some more attention is being to the issue by maternal health implementers. Accessing health extension workers nearby in the community might increase the number of ANC attendants and improve decision on the identification of health facility for delivery.

The finding of the current study showed that 175 (35%) of the respondents identified skilled birth attendants. This finding was lower than those of studies done in Indore City, India, which noted 217 (69.6%), Mpwapwa district, Tanzania 436 (86.2%), and Chamwino district, Tanzania 333 (77.8%) [11, 12, 24]. The observed discrepancy might be due to study settings, target populations, and knowledge of women about BPCR and obstetric danger signs during pregnancy, labour, and postpartum period. Conversely, it was higher than the findings of studies conducted in Adigrat and Duguna Fango district, Wolayta Zone which reported 56 (10.5%) and 62 (10.9%), respectively [17, 25]. The difference could be due to the study period, an increased number of ANC visits which might have increased access to information. The expansion of Health Extension Workers (HEWs) to the rural community might have created an opportunity for raising women’s knowledge regarding the role of skilled attendants at birth.

In the present study, saving money for delivery and any possible obstetric emergency was 336 (67.2%). This result was consistent with 388 (69%) of a study conducted in Goba woreda, Oromia Region [18]. However, our finding was lower than those of studies conducted in Indore City, India, Chamwino district, Tanzania, West Bengal, India, Mpwapwa district, Tanzania, and in Plateau State, Nigeria which reported 240 (76.9%), 360 (84.1%), 84.6%, 536 (89.3%), and 209 (83.6%), respectively [11–13, 24, 26]. This could be due to differences in settings, resulting in variations in socio-economic status, culture, and level of female empowerment. If women are empowered educationally and economically, they could have a better understanding of obstetric danger signs or complications and benefit from BPCR and save money for delivery and obstetric complication services. However, the current finding was higher than those of studies conducted in Duguna Fango district, Wolayta Zone 308 (54.1%) and Adigrat 190 (35.6%) [27, 17]. The difference might be due to variations in the study period and knowledge status of women about BPCR. The knowledge level of the respondents about BPCR was higher compared to the above studies. This fact showed that women who knew that money could help them to buy important medical supplies or pay for transportation in case of delivery and obstetrics emergency made better BPCR arrangements.

This study revealed that 202 (40.4%) of the pregnant women arranged the mode of transportation for delivery/obstetric emergency. This finding corroborated a study conducted in Adigrat and noted 40.8% [17]. However, the finding was lower compared with those of studies conducted in Mpwapwa district, Tanzania and Plateau State, Nigeria which reported 494 (82.3%) and 135 (54%), respectively [12, 13]. This could be because of variations in study settings, resulting in differences in socio-economic status and access to transportation. But our finding was higher than the result of a study (103/18.1%) done in Fango district, Wolayta Zone [27]. This could be due to an increase in the number of HEWs and repeated ANC visits which might have created awareness on the benefits of early arrangement for transportation. In cases of geographical inaccessibility or financial problems, our community arranged traditional ways of transportation such as donkeys, horses, or mules and local stretchers. Identifying transportation ahead of childbirth and improvement in decision-making can reduce maternal and neonatal mortality.

Our study found that 104 (20.8%) of the women identified potential blood donors in cases of emergency. This finding was similar to that of a study done in Plateau State, Nigeria which reported 58 (23.2%) [13]. But it was higher than the results of studies done in Fango district, Wolayta Zone 17 (3.0%), Goba woreda, Oromia Region 51 (9.1%), Robe woreda, Arsi Zone 57 (9.9%) and Adigrat 4 (0.7%) [17, 18, 21, 27]. This might be due to an improvement in the knowledge of pregnant women and their families about the importance of early identification of blood donors. In this study, nearly 100% of the pregnant women attended ANC which created a good opportunity for getting appropriate information on the elements of BPCR by skilled birth attendants. Making arrangements for blood donors is important because women giving birth may need a blood transfusion in the event of hemorrhage or cesarean section.

This study indicated that 352 (70.4%) of the pregnant women identified temporary family caregivers. This finding was lower than that of a study done in Mpwapwa district, Tanzania and documented 548 (91.3%) [12]. This could be due to differences in study settings resulting in variations in the quality of counseling and strength of applying BPCR strategy in order to reduce maternal and child mortality.

In the current study, 380 (76%) of the women identified the medical facility in preparation for the obstetric emergency. The result of this study was higher than the result of a study done in Fango district, Wolayta Zone, Goba woreda, Oromia Region, and Adigrat town, northern Ethiopia, which found out 43.6%, 47.5% and 6.6%, respectively [17, 18, 27]. This could be due to the expansion of the Health Extension Program which might have improved the number of ANC attendants in the health facilities- which probably increased awareness on obstetric danger signs and arrangements for medical facilities to cope with obstetric emergencies.

The finding showed that 346 (69.2%) of the respondents identified decision makers for cases of obstetric emergency. This finding is lower than that of a study conducted in Mpwapwa district, Tanzania, and noted 564 (94.0%) [12]. This difference might be due to variations in the socio-economic and educational status of women and the implementation of BPCR strategies. But the finding was higher than 12 (2.2%) reported from Adigrat, northern Ethiopia [17]. This could be due to differences in study periods. That is, currently the Ethiopian government has given special attention to BPCR integration and implementation at the grass-root levels by each primary health care unit and the emphasis given to male involvement in maternal health care services which might have encouraged women to identify decision-makers in cases of obstetric emergencies.

In this study, 217 (43.4%) respondents practiced BPCR. The finding was lower than the results of studies done in Indore City, India, Chamwino district, central Tanzania, Edo State, Nigeria, and the rural area of Darjeeling West Bengal, India, which reported (47.8%), (58.2%), (87.4%), and (57%), respectively [11, 24–26]. The possible reason could be the differences in study areas as the current study included both rural and urban/city communities. However, it was higher than those of studies done in rural Uganda, Aleta Wondo, southern Ethiopia, Adigrat town, northern Ethiopia, Jimma town southwest Ethiopia, Goba woreda, Oromia Region, Ethiopia, Robe woreda, Arsi zone, Oromia Region, central Ethiopia, and Dugna Fango district, Wolayta Zone, Ethiopia, which found (35%), (17%), (22.1%), (29.4%), (29.9%), (18.3%), and (16.5%), respectively [15, 17–21, 27]. The likely reason for the difference could be that the recommended BPCR elements might have been given more focus in these healthcare setups.

In the multivariate logistic regression analysis, knowledge of BPCR was two times more likely to encourage BPCR practice compared to no knowledge. This finding is inconsistent with that of a study done in Goba woreda [18]. The possible reason for the difference could be the quality of information given to women about BPCR. Increasing the number of ANC attendants and knowledge on the elements of BPCR to pregnant women during each visit might motivate them to prepare for birth and to be ready for obstetric complications.

Participants knowledgeable about danger signs during pregnancy were three times more likely to practice BPCR compared to those who had no knowledge. This finding is similar to those of studies done in Goba and Robe woredas, Ethiopia, but differs from that of a study done in Adigrat [17, 18, 21]. The possible reason could be the study period and the clarity of information on BPCR addressed at times of repeated ANC visits. Besides, their partners might have been involved in BPCR. Knowledge about the danger signs of obstetric complications is important for recognizing complications early and taking immediate actions to access essential emergency obstetric care.

Pregnant women who started their ANC services within three to four months of pregnancy were three times more likely to be prepared to practice BPCR compared with those who started late. This finding was in line with those of studies conducted in Robe woreda, Arsi Zone, Fango district, Wolayta Zone and Goba woreda [18, 21, 27]. This might indicate that there was a better exposure to information on BPCR during the repeated visits.

Rural pregnant women were 0.44 times less likely to be prepared for BPCR. That is, urban dwellers were more likely to be prepared for BPCR. This finding is similar to that of a study done in Goba woreda [18]. The possible reason could be that urban residence improves access to information, service, and BPCR practice.

Limitations of this study were since the design of our work was cross-sectional; it might not have been strong enough to demonstrate the cause and effect relationships between dependent and independent variables. In addition, as the data collectors were health professionals, there was a possibility of social desirability bias in the responses to some of the variables.

## Conclusion

The present study identified that there has been poor preparedness for birth and complications. Residence, knowledge about BPCR and danger signs during pregnancy, gestational age, and the starting time of ANC service were significantly associated with BPCR practice.

Health professionals should create awareness on birth preparedness and complication readiness among pregnant mothers, families, or communities. Government officials, partners, and health care providers that are working in areas of maternal and child health should work together to ameliorate factors that influence the low practice of birth preparedness and complication readiness. Researchers need to conduct qualitative studies to explore the main reasons for their poor preparedness of pregnant women for birth and in cases of obstetric complications in order to bring significant changes in the reduction of maternal and neonatal deaths.

## Additional files

Additional file 1: English version questionnaire. (DOCX 28 kb)

Additional file 2: Amharic version questionnaire. (DOCX 30 kb)

## Abbreviations

ANC: Antenatal care
BPCR: Birth preparedness and complication readiness
BSc: Bachelor of science
EDHS: Ethiopian demographic health survey
EFMOH: Ethiopian federal ministry of health
ETB: Ethiopian birr
FMOH: Federal ministry of health
HAD: Health development army
HEWs: Health extension workers
MDGs: Millennium development goals
MMR: Maternal mortality ratio
NGOs: Non-governmental organizations
PNC: Postnatal care
SNNP: Southern nations, nationalities, and people
SPSS: Statistical package for social sciences
TBA: Traditional birth attendant
WHO: World Health Organization
