# Supplementary material for: Birth preparedness and complication readiness among pregnant women in Tehulederie district, Northeast Ethiopia: a community-based cross-sectional study
Source: BMC Nurs. 2018 Mar 15;17:10. doi: 10.1186/s12912-018-0278-y (PMC5856365; doi:10.1186/s12912-018-0278-y)
Supplement: Supplementary file 1 — English version questionnaire. (DOCX 28 kb) [file 12912_2018_278_MOESM1_ESM.docx]

## Questionnaire on assessment of Birth Preparedness and Complication Readiness

**Part I. Socio-demographic characteristics of the respondents**

| **S.No** | **Questionnaire** | **Alternative choices** | **Skipping** |
| --- | --- | --- | --- |
| 101 | How old are you? | --------------- |  |
| 102 | What is your marital status? | 1. Single 2. Married/Union 3. Divorced 4. Widowed |  |
| 103 | What is your educational status? | 1. Not read & write 2. Read & write 3. Elementary 4. Secondary & above |  |
| 104 | Where is your place of Residence? | 1. Urban 2. Rural |  |
| 105 | What is your occupation? | 1. Housewife 2. Gov’t employee 3. Merchant 4. Pvt org. employee |  |
| 106 | How many families do you have? | ------------------- |  |
| 107 | What is your Religion? | 1. Orthodox 2. Muslim 3. Protestant 4. Catholic |  |
| 108 | What is your Ethnicity? | 1. Amhara 2. Oromo 3. Tigre 4. Afar |  |
| 109 | What is your monthly income? | ------------------ |  |

**Part II: Regarding the current pregnancy of the respondent**

| **S.No** | **Questionnaire** | **Alternative choices** | **Skipping** |
| --- | --- | --- | --- |
| 201 | How old is your current pregnancy (GA)? | 1. 3_6 months 2. 6_9 months 3. > 9 months |  |
| 202 | Did you see anyone for antenatal care for this pregnancy? | 1. Yes 2. No | Skip to Q#203 if the answer is no and to Q# 204 if the answer is YES |
| 203 | Why didn't you do so? | 1. Did not know where to go 2. Health facility is too far 3. Too expensive 4. No good services |  |
| 204 | How many ANC visits in total did you receive for this pregnancy? | 1. One time 2. Two times 3. Three times 4. Four times 5. Five times and more |  |
| 205 | If you see anyone for antenatal care, whom did you see? | 1. Doctor 2. Midwife/Clinical nurse 3. Health officer 4. HEW 5. TBA |  |
| 206 | When did you start your first ANC visit? | ----------------- |  |
| 207 | How many antenatal visits do you plan to get to this pregnancy in total? | ----------------------------------- |  |

**Part III**. **Regarding Birth Preparedness and Complication Readiness**

| **S.No** | **Questionnaire** | **Alternative choices** | **Skipping** |
| --- | --- | --- | --- |
| 301 | Have you the information about “birth preparedness and complication readiness’’ | 1. Yes 2. No | Skip to Q# 302 if the answer is YES |
| 302 | From whom did you get the information for the first time? | 1. Health professional 2. TBA 3. HEW 4. 1 to 5 leader 5. Pregnant women forum   97. Other (specify………) |  |
| 303 | Have you been counseled about different possible obstetric complications by health professionals during this pregnancy? | 1. Yes 2. No |  |
| 304 | In your opinion, what are some of the things a pregnant woman can do to be prepared for birth and to be ready for obstetric complication? | 1. Identification of health facility for place of delivery 2. Saving money for delivery and for obstetric emergency 3. Identification of skilled birth attendant 4. Identification of blood donors in case of obstetric emergency 5. Identification a means of transportation for birth or for obstetric emergency to the facility 6. Identification of birth companion 7. Identification of medical facility for obstetric emergency 8. Identification of decision-maker in case of obstetric emergency 9. Identification of temporary family caregiver in case of emergency |  |
| 305 | For your birth, which of the following things are prepared? | 1. identified health facility as a place of delivery 2. Saving money for delivery and for obstetric emergency 3. Identified a skilled birth attendant 4. Identified blood donors in case of obstetric emergency 5. Identified a means of transportation for birth or for obstetric emergency to the facility 6. Identified by birth companion 7. Identified a medical facility for obstetric emergency 8. Identified a decision-maker in case of obstetric emergency 9. Identified temporary family caregiver in case of emergency |  |

**Part IV: Regarding knowledge of key danger signs the woman**

| **S.No** | **Questionnaire** | **Alternative choices** | **Skipping** |  |
| --- | --- | --- | --- | --- |
| 401 | In your opinion, can unforeseen problems occur during pregnancy, childbirth and the postpartum period within two days that could endanger the life of a woman? | 1. Yes 2. No 3. Don’t know |  | |
| 402 | In your opinion, what are some serious health problems that can occur during pregnancy that could endanger the life of a pregnant woman? | 1. Bleeding 2. Severe headache 3. Blurred vision 4. Convulsion 5. Swollen hand/face |  | |
| 403 | In your opinion, what are some serious health problems that can occur during labor and childbirth that could endanger the life of a pregnant woman? | 1. Severe bleeding 2. Severe headache 3. Convulsion 4. High fever 5. Loss of consciousness 6. Labor lasting >12 hours 7. Baby’s hand, cord’s or its foot comes before head |  | |
| 404 | In your opinion, what are some serious health problems that can occur during the first 2 days after birth that could endanger the life of the woman? | 1. Severe bleeding 2. Convulsion 3. Blurred vision 4. Swollen of face/hand 5. Severe abdominal pain 6. High fever 7. Malodorous discharge |  | |

**Part V: Regarding the previous pregnancy of the respondent**

| **S.No** | **Questionnaire** | **Alternative choices** | **Skipping** |
| --- | --- | --- | --- |
| 501 | How many births did you give prior to the current pregnancy? | 1. 0 2. 1 3. 2 4. 3 5. 4 and above | Skip to Q#601 if the answer is ‘01’ |
| 502 | Have any of your pregnancies resulted in a baby that was born dead (a stillbirth)? | 1. Yes 2. No | Skip to Q#503 if the answer is YES |
| 503 | How many of these pregnancies resulted in a baby that was born dead? | ----------------------------------- |  |
| 504 | Have any of your pregnancies resulted in a baby that was born alive? | 1. Yes 2. No | Skip to Q#505 if the answer is YES |
| 504 | How many of these pregnancies resulted in a baby that was born alive?- | ----------------------------- |  |
| 505 | Was your most recent child be born alive or dead? | 1. Dead 2. Alive | Skip to Q #506 if the answer is alive& 601 if the answer is dead |
| 506 | In what month did you give the birth? | ----------------------------------- |  |

**Part VI**. **Health facility and services characteristics**

| **S.No** | **Questionnaire** | **Alternative choices** | **Skipping** |
| --- | --- | --- | --- |
| 601 | Do you know of a place where you can go to give birth? | 1. Yes 2. No | skip to Q#602 if the answer is YES |
| 602 | Where is that place? | 1. Respondent’s HOME 2. TBA’s Home 3. Health institutions | Skip to Q# 603 & 604 if the answer is Health institution and to Q#605 if the answer is home |
| 603 | Why you prefer this place than home? | ------------------------------------ |  |
| 604 | What is the estimated distance of the health institution from your home? | _____________ Km |  |
| 605 | Why you prefer this place than health institutions? | ---------------------------------------------------------------- |  |
| 606 | How would you go to the place where you give birth? | 1. Ambulance 2. Private car 3. taxi/ Bajaj 4. others (specify) |  |
| 607 | Can you access this mode of transportation easily? | 1. Yes 2. No |  |

**Part VII. Spouse characteristics**

| **S.No** | **Questionnaire** | **Alternative choices** | **Skipping** |
| --- | --- | --- | --- |
| 701 | What is your husband’s educational status? | 1. Not read & write 2. Read &write 3. Primary 4. Secondary & above |  |
| 702 | What is your Husband’s occupation? | 1. Farmer 2. Daily laborer 3. Gov’t. employee 4. Merchant |  |
