# Supplementary material for: Birth preparedness and complication readiness among pregnant women in Tehulederie district, Northeast Ethiopia: a community-based cross-sectional study
Source: BMC Nurs. 2018 Mar 15;17:10. doi: 10.1186/s12912-018-0278-y (PMC5856365; doi:10.1186/s12912-018-0278-y)
Supplement: Supplementary file 2 — Amharic version questionnaire. (DOCX 30 kb) [file 12912_2018_278_MOESM2_ESM.docx]

**Amharic version questionnaire on assessment of Birth Preparedness and Complication Readiness**

**ክፍል አንድ፡ ማህበራዊና ስነ-ህዝባዊ መረጃ**

1. እድሜዎ ስንት ነው? ---------- ዓመት
2. የጋብቻ ሁኔታ፡ ሀ. ያላገባች ለ. ያገባች ሐ. የፈታች መ. የሞተባት ሠ. ሌላ (ይገለፅ) ------
3. የትምህርት ደረጃዎ ሀ. ያልተማረች ለ. መፃፍ/ማንበብ ሐ. 1ኛ ደረጃ ትምህርት (1-8 ከፍል) መ. 2ኛ ደረጃ ትምህርት (9-12 ክፍል) ሠ. ኮሌጅና ከዛ በላይ
4. የመኖሪያ ቦታ ሀ. ከተማ ለ. ገጠር
5. የስራ ሁኔታ ሀ. የቤት እመቤት ለ. የመንግስት ሰራተኛ ሐ. ነጋዴ መ. የግል መ/ቤት ተቀጣሪ (መያድ፣ የግል ቢዝነስ) ሠ. ሌላ (ይገለፅ)------------
6. ስንት የቤተሰብ አባላት አለዎት? ---------------------
7. ሃይማኖት ሀ. ኦርቶዶክሰ ለ. ሙስሊም ሐ. ፕሮቴስታንት መ. ካቶሊክ ሠ. ሌላ (ይገለፅ)-------
8. ብሔርዎ ምንድ ነው? ሀ. አማራ ለ. ኦሮሞ ሐ. ትግሬ መ. አፋር ሠ. ሌላ (ይገለፅ)-------------------
9. የቤተሰብዎ ወርሃዊ ገቢ ስንት ነው? (በብር) --------------------------

**ክፍል ሁለት፡-የአሁኑን እርግዝና ሁኔታ በተመለከተ**

1. ይህን እርግዝና ካረገዝሽ ስንት ጊዜ ሁኖሻል?

ሀ. 3_6 ወር ለ. 6_9ወር ሐ. ከ9 ወር በላይ

1. የእርግዝና ክትትል አድርገሽ ታውቂያለሽ? (ለዚህ እርግዝና) ሀ. አዎ ለ .የለም

**ለጥያቄ ቁጥር 11 መልስዎ “አዎ” ከሆነ ወደ ቁጥር 13 ይለፉ**

1. የእርግዝና ክትትል አድርገው የማያውቁ ከሆኑ ለምን አድርገው አያውቁም?

ሀ. የት እንደምሄድ ስለማላውቅ ለ. ጤና ተቋሙ በጣም ሩቅ ስለሆነ ሐ. ዋጋው ውድ ስለሆነ መ. የሚሰጠው አገልግሎት ጥሩ ስላልሆነ ሠ. ሌላ(ይገለፅ)---------------------------------

1. በዚህ እርግዝና እስካሁን ድረስ ስንት ጊዜ የእርግዝና ክትትል አድርገዋል?

ሀ. አንድ ጊዜ ለ. ሁለት ጊዜ ሐ. ሶሰት ጊዜ መ. አራት ጊዜ ሠ. አምስት ጊዜና ከዛ በላይ

1. የእርግዝና ክትትል አድርገው የሚያውቁ ከሆኑ፣ ክትትሉን ማን አደረገለዎት?

ሀ. ዶክተር

ለ. አዋላጅ ነርስ/ክሊኒካል ነርስ

ሐ. ጤና መኮነን

መ. የጤን ኤክስቴንሽ ሰራተኛ

ሠ. የልምድ አዋላጅ

ሰ. .ሌላ (ይገለፅ)

1. ለዚህ እርግዝና ክትትል የጀመርሽው ባረገዝሽ በስንተኛ ወርሽ ነበር? በ ------------- ወር
2. በዚህ እርግዝና፣ እስካሁን ያደረግሽውን ክትትል ጨምሮ፣ ስንት ጊዜ የእርግዝና ክትትል ለማድረግ አስበሻል? ለ----ጊዜ

**ክፍል ሶስት፡-ለወሊድና ወሊድ መወሳሰብ ዝግጁነት እቅድ**

1. ለወሊድ ስለሚደረግ ቅድመ ዝግጅት ሰምተው ያውቃሉ? ሀ. አዎ ለ. የለም
2. ሰምተው የሚያውቁ ከሆነ መረጃውን ከይት አገኙ? **(ከአንድ በላይ መመለስ ይቻላል)**

ሀ. ከጤና ባለሙያ

ለ. ከልምድ አዋላጅ

ሐ. ከጤና ኤክስቴንሽን ሰራተኞች

መ. ከ 1 ለ 5 መሪ

ሠ. ከነፍሰጡር ኮንፈረንስ

ረ. ከሌላ (ይገለፅ)……………

1. በእርሰዎ እሳቤ አንድ ነፍሰጡር እናት ለወሊድ ከሚከተሉት የትኞቹን መዘጋጀት አለባት ብለዉ ያስባሉ? (**ከአንድ በላይ መመለስ ይቻላል)፡፡**

ሀ. የሚወልዱበትን ጤና ተቋም መምረጥ

ለ. የሚያዋልድ ጤና ባለሙያ መምረጥ

ሐ. ለወሊድ ጊዜ የሚሆን ገንዘብ መቆጠብ

መ. በወሊድ ጊዜ ትራንስፖርት ቢያስፈልግ ትራንስፖርት ማዘጋጀት

ሰ. ቤት የሚጠብቅና አብሮ የሚሄድ ሰዉ ማዘጋጀት

ረ. ደም ቢያስፈልግ ደም የሚለግስ ሰዉ ማዘጋጀት

1. እርስዎ ለወሊድ ከሚከተሉት ለየትኞቹ ዝግጅት አድርገዋል? **(ከአንድ በላይ መመለስ ይቻላል)**

1. የሚወልዱበትን ጤና ተቋም መርጠዋል? ሀ. አዎ ለ. የለም

2. የሚያዋልድ ጤና ባለሙያ መርጠዋል/አዘጋጅተዋል ወይ? ሀ. አዎ ለ. የለም

3. ለወሊድ አገልግሎት የሚሆን ገንዘብ ቆጥበዋል/አስቀምጠዋል ወይ? ሀ. አዎ ለ. የለም

4. ስንት ብር ቆጥበዋል ? ------------- ብር

5. በወሊድ ጊዜ ትራንስፖርት ቢያስፈልግ የትራንስፖርት አቅርቦት አዘጋጅተዋል ወይ? ሀ. አዎ ለ. የለም

6. ደም መለገስ ቢያስፈልግዎ፣ የሚለግስ ሰው አዘጋጅተዋል ወይ? ሀ. አዎ ለ. የለም

7. ቤት የሚጠብቅና አብሮ የሚሄድ ሰዉ መረጠዋል ሀ. አዎ ለ. የለም

**ክፍል አራት፡-ለወሊድ ያላቸውን ዝግጂት የእውቀት ሁኔታን በተመለከተ**

1. ከእርግዝና ወይም ልጅ መውለድ ጋር በተያያዘ ሊያጋጥም የሚችል ችግር አለ ብለው ያስባሉ?

ሀ. አዎ ለ. የለም ሐ. አላውቅም

1. ለጥያቄ ቁጥር 23 መልስዎ “**አዎ**” ከሆነ በእርግዝና ወቅት ህይወትን ለአደጋ የሚዳርጉ ችግሮች ምን ምን ናቸው ይላሉ? **(ከአንድ በላይ መመለስ ይቻላል)**

ሀ. የደም መፍሰስ ለ. ከፍተኛ ራስ ምታት ሐ. የእይታ መደብዘዝ

መ. መንቀጥቀጥ (የሰውነት መንዘፍዘፍ) ሰ. የእጅ/ፊት ማበጥ

1. በወሊድ ወቅት ህይወትን ለአደጋ የሚዳርጉ ችግሮች ምን ምን ናቸው ብለው ያስባሉ? **(ከአንድ በላይ መመለስ ይቻላል)**

ሀ. ከፍተኛ የደም መፍሰስ

ለ. ከፍተኛ ራስ ምታት

ሐ. መንቀጥቀጥ (የሰውነት መንዘፍዘፍ)

መ. ከፍተኛ ትኩሳት

ሰ. ራስን መሳት

ረ. ከ12 ሰዓት በላይ የፈጀ ምጥ

ሸ. የህፃኑ እጅ፣ እትብት ወይም እግር ከራሱ ቀድሞ መምጣት

1. ልጅ በተወለደ **በመጀመሪያዎቹ ሁለት ቀናት** ውስጥ የሚከሰቱ ህይወትን ለአደጋ የሚዳርጉ ችግሮች ምን ምን ናቸው ይላሉ? **(ከአንድ በላይ መመለስ ይቻላል)**

ሀ. ክፍተኛ የደም መፍሰስ ለ. መንቀጥቀጥ (የሰውነት መንዘፍዘፍ) ሐ. የእይታ መደብዘዝ መ. የእጅ/ ፊት ማበጥ ሰ. ከፍተኛ ትኩሳት ረ. ሽታ ያለው የብልት ፈሳሽ ሸ. ከፍተኛ የሆድ ቁርጠት

**ክፍል አምስት፡-የባለፈው ልጅዎን አወላለድ በተመለከተ**

1. ከዚህ እርግዝና በፊት ስንት ልጅ ወልደዋል?

ሀ. ይህ የመጀመሪያየ ነው ለ. አንድ (01) ሐ. ሁለት (02) መ. ሶስት (03) ሠ. አራትና ከዛ በላይ (≥4)

**ለጥያቄ ቁጥር 27 መልስዎ “ሀ” ከሆነ ወደ ጥያቄ ቁጥር “34” ይለፉ**

1. ከዚህ በፊት ልጅ ወልደው የሚያውቁ ከሆነ፣ ከወለዷቸው ልጆች መካከል (በማህፀን ውስጥ) “ጠፍቶ” የተወለደ ነበረ ወይ? ሀ. አዎ ለ. የለም
2. ለጥያቄ ተራ ቁጥር 28 መልስዎ “አዎ” ከሆነ ስንት ልጆች “ጠፍተው” ተወለዱ? -------------------
3. ከወለዷቸው ልጆች ውስጥ በህይዎት የተወለዱ አሉ?

ሀ. አዎ ለ. የለም

1. ለጥያቄ ተራ ቁጥር 30 መልስዎ “አዎ” ከሆነ ስንት ልጆች በህይዎት ተወለዱ? ----------------------
2. ከወለዷቸው ልጆች የመጨሻው ልጅዎ ሲወለድ እንዴት ነበር?

ሀ. ጠፍቶ የተወለደ ለ. በህይወት የተወለደ

1. ለጥያቄ ቁጥር 32 መልስዎ በህይዎት የተወለደ ከሆነ፣ የተወለደው/ችው በስንት ወሩ/ሯ ነበር? ---

**ክፍል ስድስት፡- የጤና ተቋማትና አገልግሎት ሁኔታን በተመለከተ**

1. የወሊድ አገልግሎት የት እንደሚሰጥ ያውቃሉ? ሀ. አዎ ለ. የለም
2. ለጥያቄ ቁጥር 34 መልሰዎ “አዎ” ከሆነ፣ በእርስዎ ግምት የወሊድ አገልግሎት መሰጠት ያለበት የት ቦታ ነው ይላሉ? ሀ. እቤቴ ለ. የልምድ አዋላጇ ቤት ሐ. ጤና ተቋማት መ. ሌላ (ካለ ይገለፅ)
3. ለጥያቄ ተራ ቁጥር 35 መልስዎ “ጤና ተቋም” ከሆነ ይህን ቦታ ለምን መረጡት? **(በዝርዝር ይንገሩን) ---------------------------------------------------------------------------------------------------------------------------------------------------------------------------------------------**
4. የወሊድ አገልግሎት የሚሰጥበት ጤና ተቋም ከእርስዎ ቤት ምን ያህል ይርቃል? (በሜትር ወይም በኪ/ሜትር ግለፁ) -------------------
5. ለጥያቄ ቁጥር 35 መልስዎ “ቤት” ከሆነ ይህን ቦታ ለምን መረጡት? -------------------------------------------------------------------------------------------------------------------------------------------------------------------------------------------------------------------------------------------------------------
6. ከእርስዎ ቤት ወደ ጤና ተቋሙ ለመሄድ ምን ዓይነት የትራንስፖርት አገልግሎት አለ?

ሀ. አንቡላንስ

ለ. ታክሲ/ባጃጅ

ሐ. የግል መኪና

መ. ሌላ (ካለ ይገለፅ)

1. የትራንስፖርት አገልግሎቱን በቀላሉ ያገኙታል ወይ? ሀ. አዎ ለ. የለም

**ክፍል ሰባት፡-የባል/የትዳር አጋርን ሁኔታ በተመለከተ**

1. የባለቤት/ባልሽ የትምህርት ደረጃ ስንት ነው? ሀ. ያልተማረ ለ. መፃፍ/ማንበብ የሚችል ሐ. 1ኛ ደረጃ ትምህርት (1-8 ከፍል) መ. 2ኛ ደረጃ ትምህርት (9-12 ክፍል) ሠ. ኮሌጅና ከዛ በላይ
2. የባለቤትሽ/ባልሽ ስራው ምንድነው ? ሀ. ገበሬ ለ. የቀን ሰራተኛ ሐ. የመ/ሰራተኛ መ. ነጋዴ ሠ. ሌላ (ይገለፅ) ----------------------

**ይህ የመጠይቁ ማብቂያ ነው እናመሰግናለን!**

የቃለ መጠይቁ መረጃ የተሰበሰበበት ቀን ፡- -----------------------------

መረጃውን የሰበሰበው ግለሰብ ስም ፡- ---------------------------------

ፊርማ ፡- ---------------------------------

የተቆጣጣሪ ስም ፡- -------------------------------

ፊርማ ፡- ------------------------------
